# Feasibility of source-free DAS logging for next-generation borehole imaging

**DOI:** 10.1038/s41598-022-16027-3

**Published:** 2022-07-13

**Authors:** David Li, Lianjie Huang, Yingcai Zheng, Yingping Li, Philip Wannamaker, Joseph Moore

**Affiliations:** 1 Geophysics Group, Los Alamos National Laboratory, MS D452, Los Alamos, NM 87545 USA; 2 Department of Earth & Atmospheric Sciences, University of Houston, Houston, TX 77004 USA; 3 BlueSkyDas LLC, Sugar Land, TX 77479 USA; 4 Energy & Geoscience Institute, University of Utah, 423 Wakara Way, Salt Lake City, UT 84108 USA

**Keywords:** Solid Earth sciences, Geophysics

## Abstract

Characterizing and monitoring geologic formations around a borehole are crucial for energy and environmental applications. However, conventional wireline sonic logging usually cannot be used in high-temperature environments nor is the tool feasible for long-term monitoring. We introduce and evaluate the feasibility of a source-free distributed-acoustic-sensing (DAS) logging method based on borehole DAS ambient noise. Our new logging method provides a next-generation borehole imaging tool. The tool is source free because it uses ever-present ambient noises as sources and does not need a borehole sonic source that cannot be easily re-inserted into a borehole after well completion for time-lapse monitoring. The receivers of our source-free DAS logging tool are fiber optic cables cemented behind casing, enabling logging in harsh, high-temperature environments, and eliminating the receiver repeatability issue of conventional wireline sonic logging for time-lapse monitoring. We analyze a borehole DAS ambient noise dataset to obtain root-mean-squares (RMS) amplitudes and use these amplitudes to infer subsurface elastic properties. We find that the ambient noise RMS amplitudes correlate well with anomalies in conventional logging data. The source-free DAS logging tool can advance our ability to characterize and monitor subsurface geologic formations in an efficient and cost-effective manner, particularly in high-temperature environments such as geothermal reservoirs. Further validation of the source-free DAS logging method using other borehole DAS ambient noise data would enable the new logging tool for wider applications.

## Introduction

Many energy and environmental applications require reliable characterization and monitoring of geologic formations around a borehole. In enhanced geothermal systems (EGS) and unconventional oil/gas, characterizing near borehole geologic formations is essential for effective fracture stimulation to extract geothermal energy and oil/gas, respectively. Wellbore integrity monitoring is crucial for both EGS and geologic carbon storage.

Conventional wireline sonic logging tools are extensively used to measure physical properties of geologic formations around a borehole, but they have limitations in long-term borehole monitoring. Typically, a conventional sonic logging tool (~ 3 m in length) consists of a receiver array containing multiple levels of sensors and one or more acoustic sources (monopole, dipole, or quadrupole) that emit P-, S-, Stoneley, and flexural borehole waves to estimate local formation velocities or to image near borehole geologic formations^1,2^. Sonic logging tools require a fluid filled borehole to operate and produce large-amplitude dispersive borehole waves along the surface of the fluid fill borehole that often make data processing and imaging difficult^3^. Like other wireline logging tools, sonic logging tools cannot operate in harsh, high-temperature environments (> 150 °C) for a long period of time. However, monitoring time-lapse changes requires repeated measurements over the reservoir life. The difficulty of re-inserting wireline logging tools into a borehole after well completion makes long-term monitoring either difficult, economically infeasible, or even impossible.

We introduce an innovative source-free distributed-acoustic-sensing (DAS) logging method based on borehole DAS ambient noise. Because the acquisition instrument for DAS is a fiber optic cable, the new logging tool can be deployed in harsh environments to continuously acquire data with a dense spatial sampling (1 m) along the entire length of the borehole. Compared with conventional logging instruments, the fiber optic cable does not contain sensitive electronics and can be installed in the borehole permanently to collect data over long periods of time. The durable nature of optical fibers in harsh, high temperature and pressure environments, has allowed DAS and Distributed Temperature Sensing (DTS) systems to become favored down-hole tools to explore, characterize, and monitor unconventional and geothermal energy resources. Therefore, many surface DAS ambient noise studies have been conducted. For example, ambient noises recorded by surface DAS systems have been studied and are widely used to derive shallow S-wave velocity structures and detect underground water^4–12^. However, few studies on borehole DAS ambient noise characteristics along depth have been reported^13,14^.

We evaluate the feasibility of our source-free DAS logging method by analyzing a borehole DAS ambient noise dataset acquired at the Frontier Observatory for Research in Geothermal Energy (FORGE) site in Utah. The Utah FORGE site is a field laboratory used to test and develop EGS technologies. We analyze borehole DAS ambient noise data collected within the granitic basement rock using a 1-km optic fiber cable installed in cement behind a cased vertical observation well. We calculate the root-mean-squares (RMS) amplitudes of borehole DAS ambient noise and construct a depth profile of ambient noise RMS amplitudes. We find that the noise RMS amplitude constantly varies with depth, showing peaks at several distinct depths. We compare the noise RMS depth profile with available wireline logging data in both the injection and the observation wells and find apparent correlations between major RMS peaks and low-velocity layers (LVL) bounded by sharp structural interfaces. We also find that the noise RMS amplitude peak zones only correlate with LVLs, which also correspond to associated depths with both low Poisson’s ratio and high porosity, indicating highly fractured zones. Therefore, we can use borehole DAS ambient noise data as sonic logging data to potentially locate fractured zones in the borehole.

## Results

### Borehole DAS observation system at the Utah FORGE site

**Figure 1:**
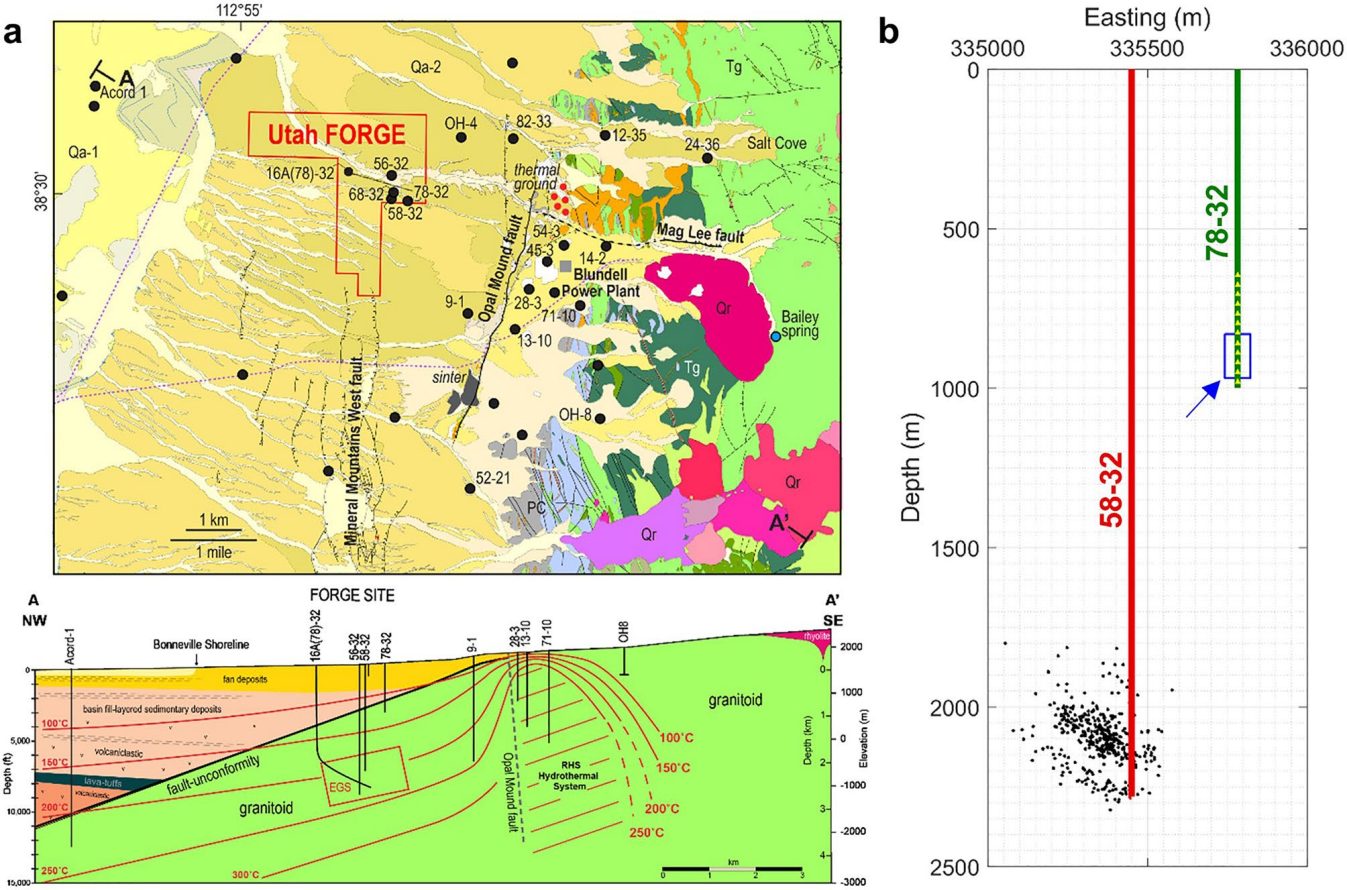
Geologic maps and cross-sections of the Utah FORGE site. (**a**) Geologic map and cross-section of the Utah FORGE geothermal site (modified from Kirby et al.^17^ by courtesy of The Utah Geological Survey). The treatment well (58–32) was drilled through fan and sediment deposits and reached the geothermal reservoir (EGS) in the low permeability granite rocks at a depth of 2294 m with a temperature of about 200 °C. The observation well (78–32) was drilled to a depth of 1000 m. A fiber-optic cable and 12 three-component (3C) geophones were deployed in well 78–32 to monitor activities in the treatment well. (**b**) A zoomed in view of the treatment well 58–32 (red line) and monitoring well 78–32 (green line). The black dots are the location of microseismic events, the yellow dots show the 12 3-C geophone locations, and the blue box shows the area of investigation for this study. Both the treatment and monitoring wells are nearly vertical.

The Utah FORGE geothermal site in Beaver County, Utah is a field laboratory for EGS development and research^15,16^. Figure 1a shows a map view and a cross section of the Utah FORGE site modified from Kirby et al.^17^ by courtesy of the Utah Geological Survey. Well 58–32 is a near-vertical well drilled to a depth of approximately 2297 m to characterize an EGS fracture stimulation zone in the granitic basement host rock. Well 78–32 is a near-vertical monitoring well with a depth of approximately 1000 m.

Between April 19 and May 3, 2019 during Phase 2C of the FORGE project, a borehole array of twelve 3C geophones and a DAS system were deployed in observation well 78–32 to monitor induced microseismic events during stimulation cycles in treatment well 58–32^16^. Figure 1b is a cross-section displaying treatment well 58–32, monitoring well 78–32, and induced microseismic events (small black dots). The distance between the two wells is approximately 336 m. A 1-km optic fiber was installed in the observation well (78–32) to record DAS data with a channel spacing of 1 m, gauge length of 10 m, and time sampling rate of 0.5 ms.

The cross-section in Fig. 1b depicts the locations of the monitoring well, treatment well, and a 12-level 3C geophone string (yellow dots). The Carina DAS system continuously recorded data including ambient noise from April 19 to May 3 during a series of stimulation experiments in the treatment well 58–32.

### Depth profiles of RMS amplitudes of borehole DAS ambient noise

**Figure 2:**
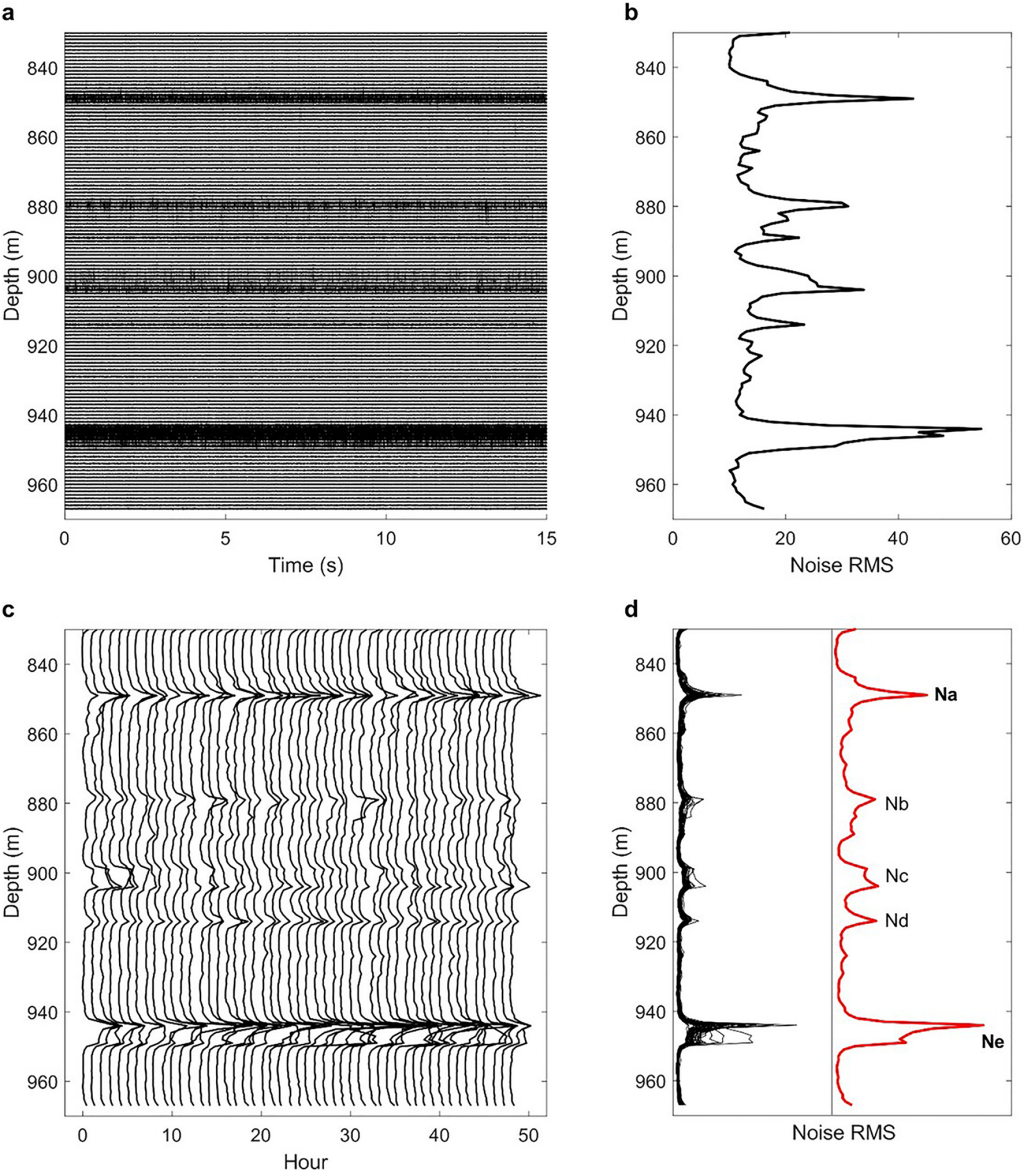
DAS ambient noise data and noise RMS depth profiles. (**a**) Fifteen-second ambient noise traces of 138 DAS channels ranging from depths of 830 to 968 m. (**b**) Noise RMS depth profile showing noise RMS amplitudes varying with depths. (**c**) Ambient noise RMS amplitude depth profiles for each hour in a 48-h period. (**d**) 48 RMS depth profiles of ambient noise superimposed on the left showing consistent features with a few exceptions. The red curve on the right is the RMS depth profile averaged over the 48-h period. There are consistent noise peaks at certain depths, marked Na, Nb, Nc, Nd, and Ne. This average RMS profile indicates noise RMS amplitude levels vary with depth with several distinct peaks. The profile of the red curve is similar to that in (**b**). Note that the amplitude scale of the red curve is not the same as that of the black curves for easy identification of Nb to Nd.

We analyze the borehole DAS ambient noise recorded at depths between 830 and 968 m (channels 1016 to 1153) near the bottom of monitoring well 78–32 inside the granitoid basement as indicated by the blue rectangle in Fig. 1b. From the 14 days of continuous DAS recordings, we analyze only the borehole DAS ambient noise data in a 48-h period between April 29 and May 1, 2019, to avoid the most active stimulation cycles in the treatment well (58–32) and obtain depth profiles of RMS amplitudes of borehole DAS ambient noise. Figure 2a displays a 15-s record of the borehole DAS ambient noise data for channels 1016–1153, showing that some channels at distinct depths exhibit strong ambient noise.

To quantitatively measure the ambient noise strength of a DAS channel in a given time window with a single value, we calculate the RMS amplitudes on 15-s traces on all channels from depths of 830 to 968 m (Fig. 2a) and construct a depth profile of ambient-noise RMS amplitudes (Fig. 2b). The depth profile shows that the ambient-noise RMS amplitudes vary with depths with amplitude peaks at a few distinct depths.

We analyze one 15-s window of noise data for each hour and calculate the ambient noise RMS amplitudes for 49 15-s DAS record segments to construct 49 depth profiles of noise RMS amplitudes (Fig. 2c). These RMS depth profiles are similar. We display a superimposed plot of 48-h RMS depth profiles in the left column of Fig. 2d. These depth profiles show a high degree of similarity with one another. We average them and show the average RMS amplitude depth profile as the red curve in the right column of Fig. 2d. Note that the average RMS profile in Fig. 2d is almost identical to the curve in Fig. 2b. This observation confirms that the depth variations of noise RMS amplitudes are true and keep a consistent pattern over time.

### Correlating noise RMS amplitude profiles with borehole logging data

**Figure 3:**
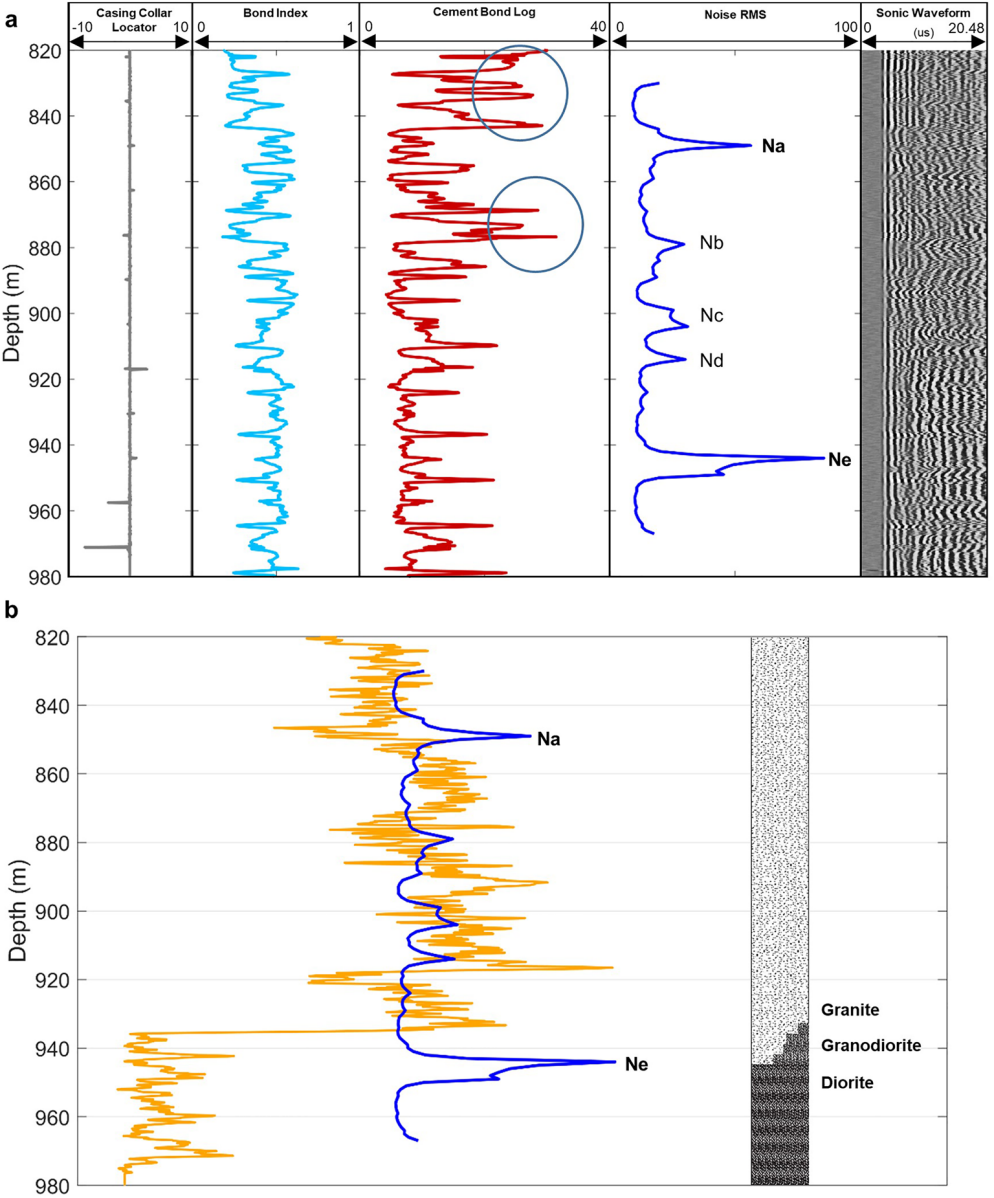
Noise RMS depth profile compared with well 78–32 logging data. (**a**) Ambient noise RMS depth profile (thick blue curve) plotted with casing collar locator (CCL), bond index (BI), cement bond log amplitude (CBLA), and sonic waveforms (right column). Clear first and later arrivals on the sonic waveforms indicate the casing and cementing are in good condition. The major peaks of noise RMS amplitudes show no correlation with the relative high amplitudes (indicated by blue circles) of the CBLA curve nor with the casing collar locator (CCL). (**b**) Ambient noise RMS depth profile (thick blue curve) versus gamma ray log curve (orange curve) and lithological log (right column). A major RMS peak (Na) at a depth of 849 m, correlates with a trough of the gamma ray curve. The major RMS peak (Ne) at a depth of 945 m coincides with the interface between granite and diorite on the lithological log.

To understand the physical meaning of the noise RMS peaks at depths, we compare the noise RMS depth profile with the cased-hole Cement Bond Log (CBL) dataset, including sonic waveforms, casing collar locator (CCL), bound index (BI), and cement bond log amplitude (CBLA), acquired in the monitoring well 78–32. We compare the noise RMS amplitude curve (thick blue line) with the CBL logging curves and sonic waveforms in Fig. 3a. Amplitudes of the first arrivals in the sonic data and late reflections of the sonic waveforms indicate that the casing and borehole cementing are both in good condition. We do not find correlation between the CCL and BI curves with the RMS curve. Large values in the CBLA curve typically represent poor quality casing/cementing. However, the peak values of CBLA (red curve in Fig. 3a) do not correspond to any of the noise RMS peaks. Therefore, the hypothesis of a relationship between RMS peaks and the casing/cement quality is not supported by the CBL data and we rule it out.

We then compare the noise RMS profile with the cased-hole gamma ray log (Gr) curve acquired in the monitoring well (78–32), as shown in Fig. 3b. Gamma ray logging measures radioactivity in rocks around a borehole to derive lithology. Sudden changes in the gamma ray values typically indicate boundaries between different formations with different radioactive contents. Figure 3b shows that the shallow noise zone around peak Na in the RMS curve (at about 849 m depth) matches perfectly with an apparent trough in the gamma ray curve. We observe that two sharp peaks in the gamma ray curve occur near the noisy zone around peak Ne in the RMS curve at a depth of about 945 m. We also observe a sudden decrease of the gamma ray value just above this noisy zone. Because the casing/cementing of the well might obstruct cased-hole Gr logging, we should use the cased-hole logging data with caution to avoid over interpretation of the data.

We also compare our results with a lithological log from a drilling report of the monitoring well, as shown in the right column in Fig. 3b. The peak Ne on the RMS profile (thick blue curve) coincides with the boundary between the granodiorite and the diorite shown on the lithological log (Fig. 3b). The position of a sudden drop in the gamma ray value is consistent with a boundary between the granite and the granodiorite. These finding of correlations between the noise RMS peaks and interfaces between different formations demonstrate the feasibility of using borehole DAS ambient noise as a source-free DAS logging tool.

**Figure 4:**
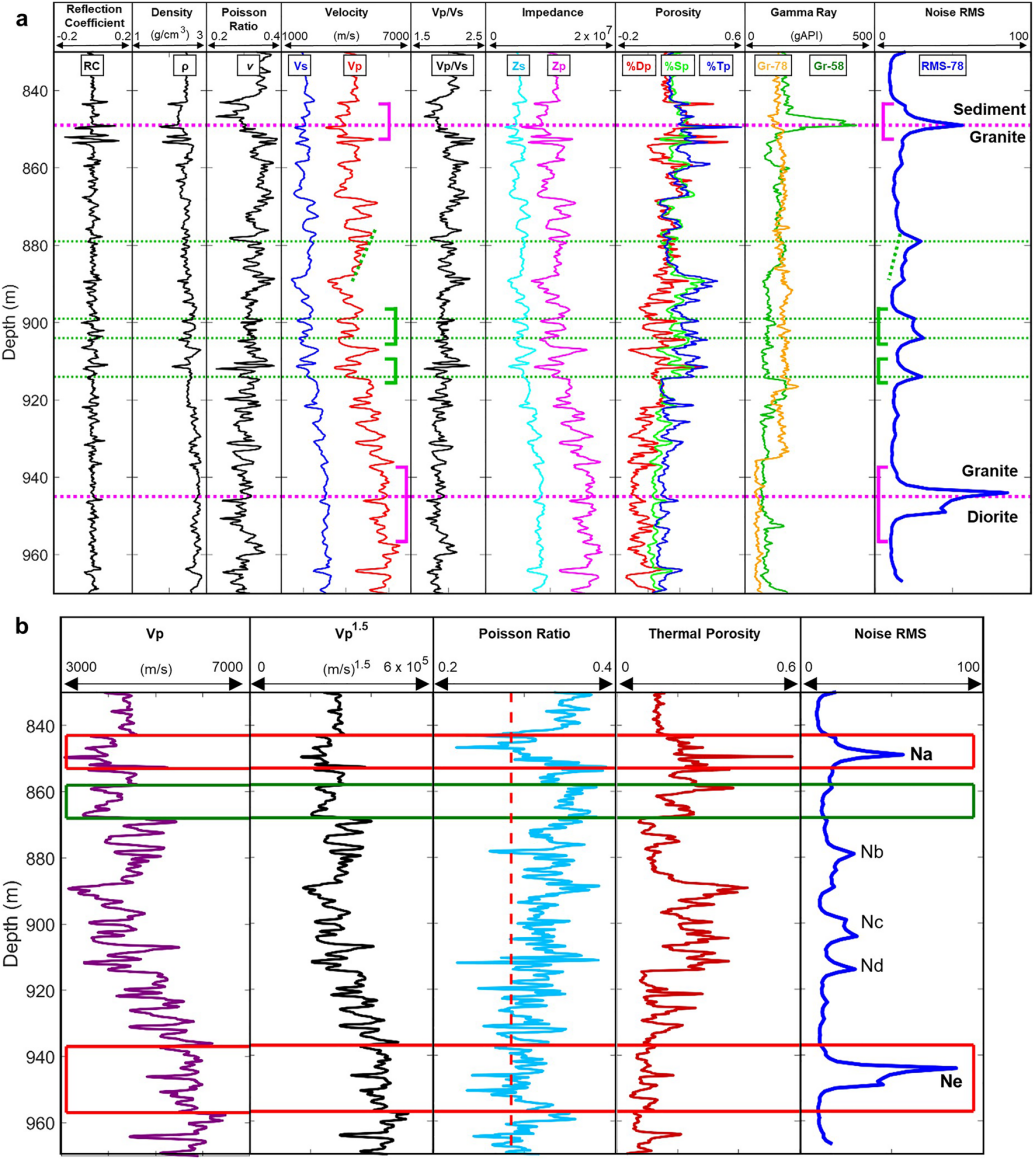
Noise RMS depth profile compared with well 58–32 logging data after depth correction. (**a**) Noise RMS profile (right column) compared with P-wave reflection coefficient (RC), density (ρ), Poisson’s ratio (ν), S-wave velocity (Vs), P-wave velocity (Vp), S-impedance (Zs), P-impedance (Zp), density porosity (%Dp), sonic derived porosity (%Sp), and thermal neutron porosity (%Tp), gamma ray (Gr-58), and well 78–32 gamma ray (Gr-78). We shift the depth of 58–32 logs upwards by 124.5 m to account for a 20° formation dip. The magenta brackets show depths of major noise zones (849 and 945 m) that correspond to thicker LVZs bounded by sharp interfaces. The horizontal dashed magenta lines show the major noise peaks, corresponding to ρ troughs, porosity peaks, distinct RCs, and the location of the sediment-granite boundary and granite-diorite boundaries. The green brackets show three weaker noise zones that correlate with LVLs with less sharp boundaries. The peaks (green dashed lines) of these noise zones also correspond with thin LVLs within the thick LVZs. The dashed green line at 876–889 m shows a gradient on both the RMS and Vp profiles. The slight shift between the noise RMS amplitude peaks and Vp troughs may be attributed to depth uncertainties between the wireline cable and DAS fiber. (**b**) Noise RMS profile compared with P-wave velocity (Vp), P-wave velocity to the power of 1.5 (Vp^1.5^), Poisson’s Ratio, and thermal porosity. The depths marked by the red rectangles, 834–853 m (Na) and 937–957 m (Ne), show a consistent pattern of low velocities, low Poisson ratio, high porosities, and large noise RMS values, indicating highly fracturing LVL. The red vertical dashed line at Pr = 2/7 indicates the transition from brittle to ductile regimes. For the Pr < 0.25 region, rocks are easier to fracture. At depths from 859 to 868 m (marked by green rectangular), there is an LVL with high porosity, but a high Poisson ratio of about 0.35 in a ductile regime, which corresponds to a quiet zone of the noise RMS profile.

Additional open-hole wireline measurements in well 58–32 can be used to understand the correlations between the noise RMS pattern and the physical properties of formations bounded by interfaces. Figure 4a shows calculated P-wave reflection coefficients (RC), density (ρ), ratio of P and S velocities (Vp/Vs), Poisson’s ratio (ν), S-wave impedance (Zs), P-wave impedance (Zp), S-wave velocity (Vs), P-wave velocity (Vp), density porosity (%Dp), sonic derived porosity (%Sp), thermal neutron porosity (%Tp), and gamma ray (Gr, green line). We compare these logs with the gamma ray (Gr-78, orange line) and the noise RMS depth profile (RMS-78, blue line) of the monitoring well (78–32). The depth in Fig. 4 is the measured depth relative to the Kelly Bushing (KB) of the monitoring well 78–32. Because there is a 20° dip in the geological formation, we subtract 124.5 m from the measured depth of the treatment well 58–32 logging data to account for this dip.

We find that the noisy zone within the upper magenta bracket with a noise RMS amplitude peak at 849 m perfectly coincides with a 10-m-thick low-velocity zone (LVZ1) in the P-wave velocity (Fig. 4a). The steep and sharp boundaries are evidenced by quick changes in density, velocities, impedances, and strong reflection coefficients at the boundary interfaces. LVZ1 contains four thin low-velocity layers (LVLs), each with a thickness of only a few meters. Among these thin LVLs, the sharpest and thinnest LVL (849 m) has a thickness of 1.1 m with the minimum P-wave velocity of 3068 m/s.

The noisy zone within the lower magenta bracket with an RMS amplitude peak at a depth of about 945 m in Fig. 4a correlates well with the other P-wave low-velocity zone (LVZ2) with a total thickness of 20 m. LVZ2 contains about five to six thin LVLs. The thinnest of these LVLs has a thickness of 2.7 m with a minimum P-wave velocity of 4770 m/s. The remaining three noisy zones with small RSM amplitude peaks in the middle (~ 879 m, 904 m, and 914 m) of Fig. 4a also correspond reasonably well to low-velocity zones (marked by green brackets). However, the boundaries of these LVLs are less sharp. The two noisiest zones with the major RMS amplitude peaks at depths of 849 m and 945 m match well with the two LVZs bound by sharp boundaries.

We focus on the thinnest, sharpest LVL with an extremely low P-wave velocity of 3068 m/s at a depth of about 849 m within LVZ1 (Fig. 4a). This thin LVL corresponds to a high porosity peak that occurs at the peak of noise RMS amplitudes (thick blue curve) in the noisy zone at depths between 843 and 853 m. Similarly, a thin, sharp LVL with the minimum P-wave velocity of 4770 m/s and high porosities at a depth of 945 m, within the 20-m-thick LVZ2, coincides with the depth of the peak of noise RMS amplitudes (thick blue curve), within the noisy zone between a depth of 937 and 957 m (Fig. 4a). The two major noisy zones (e.g., Na and Ne in Fig. 2d) shown by the noise RMS profile (thick blue curve in Fig. 4a) match well with the two LVZs (marked by the two magenta brackets). The two sharp, thin LVLs within the LVZs coincide with the two major peaks of the RMS amplitude profile. We interpret the two major noisy zones on the RMS profile as two major fault zones, containing fine structures of thin LVLs. We find that the two RMS peaks delineate boundary interface between sediment and granite and between granite and diorite.

We find that the four peaks of three secondary noisy zones between 875 and 918 m in depth with smaller RMS amplitudes (the green lines in Fig. 4a) compared with the two major noise zones, also match reasonably well with the corresponding thin LVLs indicated by the P-wave velocity curve (red line). We note that the two peaks of the noise RMS profile (thick blue line) at depths of 880 m and 914 m slightly shift from the troughs of the P-wave velocity curve of the corresponding LVLs (green dash lines). This slight shift may reflect measured depth uncertainties in either the wireline logging, or the installed DAS cable, or both. We interpret that the noise peaks with smaller RMS amplitudes seem to relate with secondary minor faults or interfaces within the granite rocks.

## Discussions

Our findings indicate that the current DAS system (1-m spatial resolution) can identify and detect major faults/interfaces and LVLs in subsurface geological formations and monitor activities of geological faults and interfaces. A DAS system with a channel spacing of 0.2 to 0.25 m^18–20^ and high time sampling rate (~ 0.05 ms) can be used as for a source-free DAS logging tool based on borehole DAS ambient noise. This DAS logging tool can also be used with conventional sonic logging tools using a sonic source to calibrate the DAS cable depth and image faults and fractures around the borehole using single-well imaging (SWI) methods^3,21^.

One possible cause of the large noise RMS amplitudes in the noisy zones is the guided waves trapped in LVLs, similar to large-amplitude surface waves trapped in low-velocity fault zones recorded by seismometer arrays^22–26^ or DAS systems^27,28^ DAS systems record the strain rate (sensitive to Vp^1.5^) instead of the particle velocity by seismometers^29–31^, resulting in high sensitivity of DAS systems to velocity drops^32^.

The two major zones in the noise RMS profile correspond with the zones with higher thermal porosities and low Poisson ratios. High porosities in granitic rocks are localized in faults, fractures, and adjacent damage zones characterized by elevated fracture density or in volumes altered by hydrothermal processes^33^. Granite porosity can be attributed to a significant increase caused by fracturing and alteration^33^. In our case, high porosities in the two zones may be attributed to fracturing in the LVLs. The dashed red line in the Poisson’s ratio plot in Fig. 4b indicates brittle and ductile transition at Pr = 2/7 (0.286) based on theoretical and experimental studies in rock mechanics^34–38^. A low Poisson’s ratio (0.1–0.25) means that rocks fracture easily whereas a high Poisson’s ratio (0.35–0.45) means that rocks are harder to fracture^39^. The rocks in the two LVL zones (Fig. 4) that have Poisson ratios less than 0.25 are in brittle regime and easy to fracture. We note that there is an apparent LVL with high porosities between depths of 859–868 m. This LVL corresponds to a quiet zone in the noise RMS amplitude curve. The rocks in this depth zone are in a ductile regime with high Poisson ratio of about 0.35 and are hard to fracture. Therefore, our noise RMS amplitude profile provides a means to detect highly fractured LVLs with major interfaces or faults. The RMS noisy zones we identified allow us to optimally select the treatment positions for effective fracture stimulation.

The source-free DAS logging tool based on ambient noise can also be used to monitor wellbore integrity. Fractures around the wellbore pose a significant risk to wellbore integrity. Our source-free DAS logging tool can directly identify and monitor geological fractures. Another advantage of our DAS logging tool is that it can be used after well completion because the DAS fiber cable is cemented behind casing. Because the fiber is permanently installed, it can provide long-term monitoring without active sources. This source-free DAS logging tool can continually survey well integrity by monitoring for vibrations and flow without disrupting well activities. Because the optic fiber cable is cemented behind casing, the source-free DAS logging can also monitor for cement and casing damage over time.

## Methods

### Classifying and processing borehole DAS ambient noise

**Figure 5:**
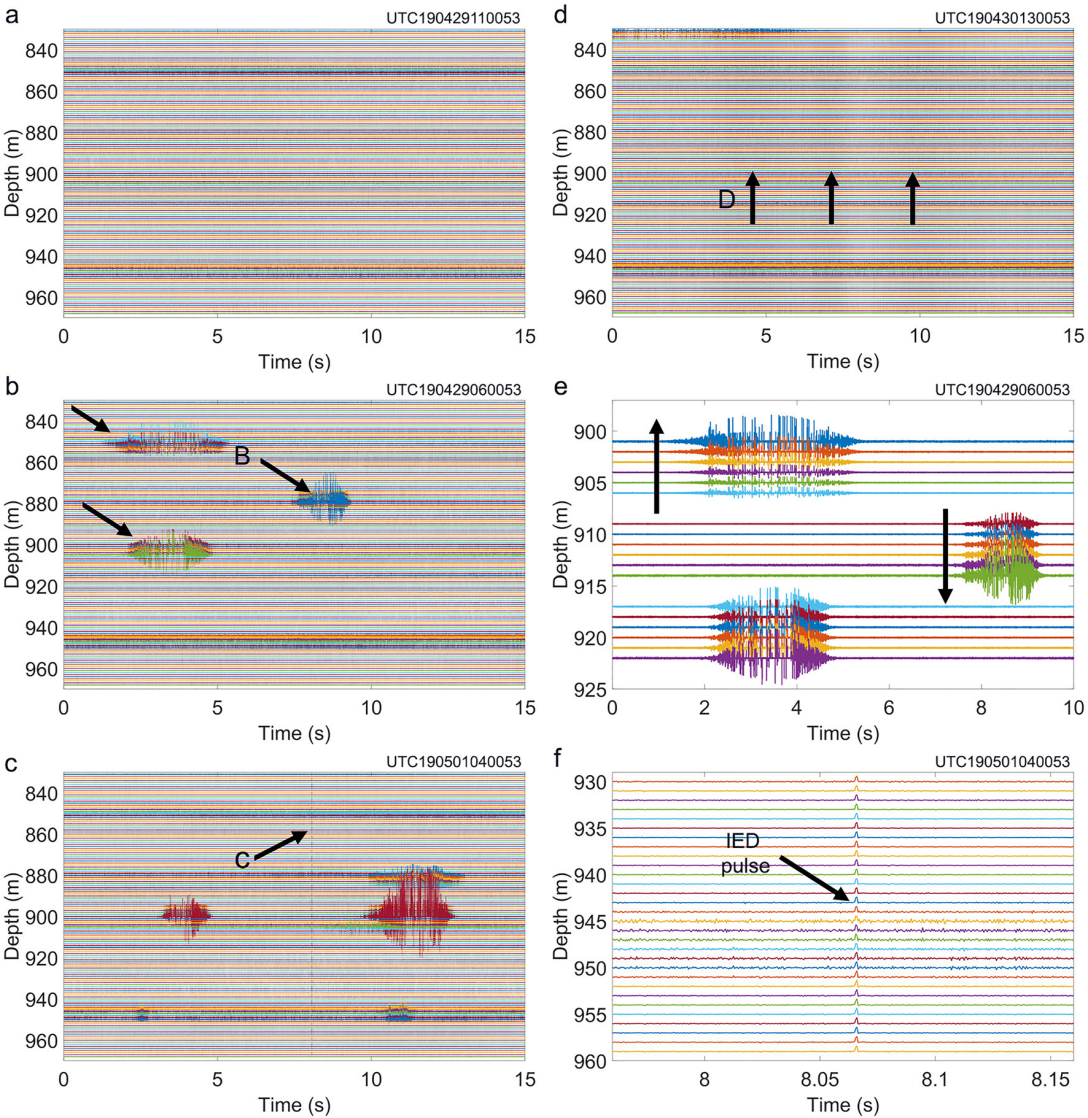
Types of noises recorded by DAS system. (**a**) Ambient noise. (**b**) Type B noise, six events within 5 m (6E5M). (**c**) Type C noise, caused by interrogator end disturbance (IED) that appears on all DAS channels simultaneously. (**d**) Noise caused by a suspected local or regional seismic event. (**e**) Zoomed in view of three groups of Type B noise (6E5M). Note that 6E5M noise amplitudes vary with channel depths. They either increase or decrease with the increasing channel depth. (**f**) Enlarged plot of Type C noise (IED) showing a simple pulse on all DAS channels at the same time.

We first define the useful “ambient noise” (Type A noise) studied and classify other types of undesirable noise excluded in our analysis of borehole DAS ambient noise. Figure 5a shows seismograms for typical 15-s noise records. In Fig. 5, the vertical axes are the depths of the DAS channels. The code on the upper right corner in each panel in Fig. 5 is the data ID number. The amplitude of ambient noise at each channel in Fig. 5a varies with depth, showing a pattern of alternating noisy and quiet zones.

Figure 5b displays the “Type B” noise. There are three time–space groups (black arrows) shown on the plot and each group consists of 6 events in a depth interval of 5 m (6E5M). We are not sure what the origin of this noise is. However, because Type B noise occurs in a consistent pattern, this noise likely comes from a non-natural origin. Type C noise (Fig. 5c) originates from the DAS interrogator end disturbance (IED) with a simple pulse or multiple pulses. Figure 5f exhibits that the IED pulses appear on all channels with little time move outs. Type D noise (Fig. 5d) seems to come from a “local” or “regional” earthquake with distinct arrivals of seismic phases. However, we have not found an earthquake catalog to confirm such an event. Fortunately, such seismic events are rarely observed and only appeared once in the 48-h time period of data that we analyze. We exclude Types B, C, and D noise from our ambient noise analysis.

An enlarged plot (Fig. 5e) reveals details for Type B noise (6E5M). This noise always appears with six events in a group; however, there are variations of noise amplitudes between channels in each group. The bottom six traces in Fig. 5e show that the noise amplitudes of each channel are comparable. However, the top and middle six traces indicate that noise amplitudes of Type B noise either increase or decrease with increasing the channel depths. Figure 5f shows that Type C noises are simple negative pulses with pulse widths of 2 ms and have similar amplitudes. This plot also shows an enlarged version of ambient noise traces in noisy and quiet zones. Because the pulses of Type C noise arrive simultaneously across all channels, we apply a simple method to remove Type C noise and recover the ambient noises. For Types B and D noises, we simply mute them and exclude them from further analyses.

**Figure 6:**
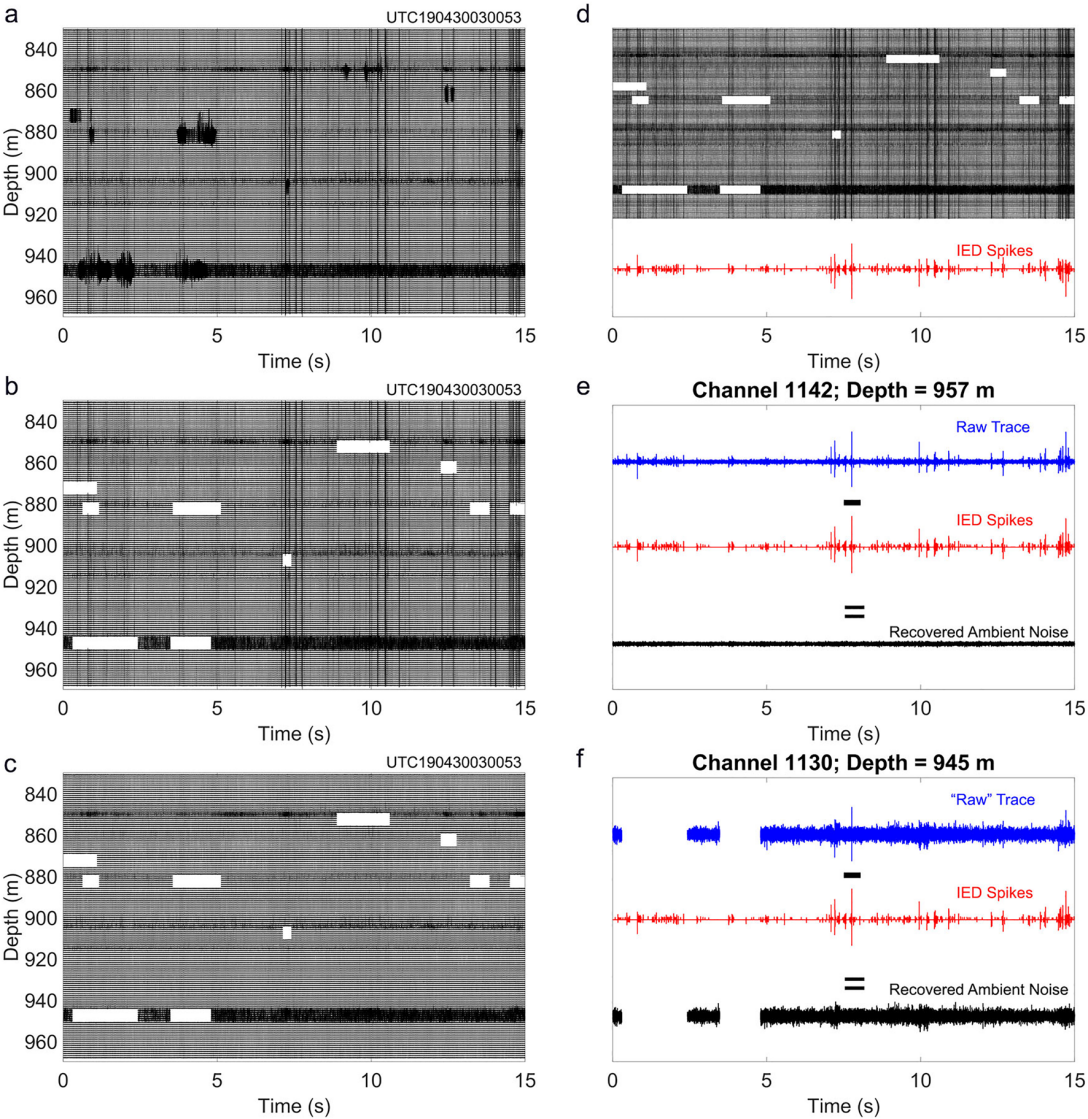
Workflow to remove Types B and C noises from the ambient noise and de-spiking procedure to remove Type C noise. (**a**) Raw 15-s-long noise traces with Types B and C noises. (**b**) Type B noise removed. (**c**) Type C noise removed using a de-spiking method. (**d**) 15-s noise traces of all channels are stacked and averaged to form a trace of IED spikes. (**e**) The trace of IED spikes is subtracted from the raw noise trace to recover ambient noise of a relatively quiet channel (1142) at a depth of 957 m. (**f**) Recovered useful ambient noise trace of a relatively noisy channel (1130) at a depth of 945 m.

We show an example of a 15 s DAS record in Fig. 6a–c to illustrate a procedure to remove Type B (Fig. 6b) and Type C (Fig. 6c) noises and recover “pure ambient noise” from raw data (Fig. 6a). We first mute 10 groups of Type B noise (6E5M) (Fig. 6b), and then employ a de-spiking method to remove Type C noises to recover ambient noise (Fig. 6c). We stack the traces of all channels in Fig. 6b after removing Type B noise to obtain a time series of IED spikes (red line in Fig. 6d). We subtract the curve of IED spikes from the raw DAS data of each channel. Figure 6e and f show examples of recovered ambient noise traces for a quiet channel and a noisy channel, respectively.

To quantitively measure the ambient noise strength of a DAS channel in a given time window with a single value, we calculate a root-mean-squares (RMS) amplitude for ambient noises recorded by the DAS system using:

$$RMS=\sqrt{\frac{1}{n}\sum_{i}{y}_{i}^{2}}$$

where *y* is the amplitude of ambient noise and *n* is the number of samples in a time series.

We calculate the RMS amplitudes of ambient noise on 15 s traces for all channels from depths of 830 to 968 m (Fig. 2a) and construct a RMS amplitude depth profile (Fig. 2b) of ambient noise recorded by the DAS system in well 78–32 at the Utah FORGE site. The depth profile of the ambient noise RMS amplitudes indicates that RMS amplitudes vary with depths, showing amplitude peaks at a few distinct depths.

### Analyzing depth profile of noise RMS amplitudes

**Figure 7:**
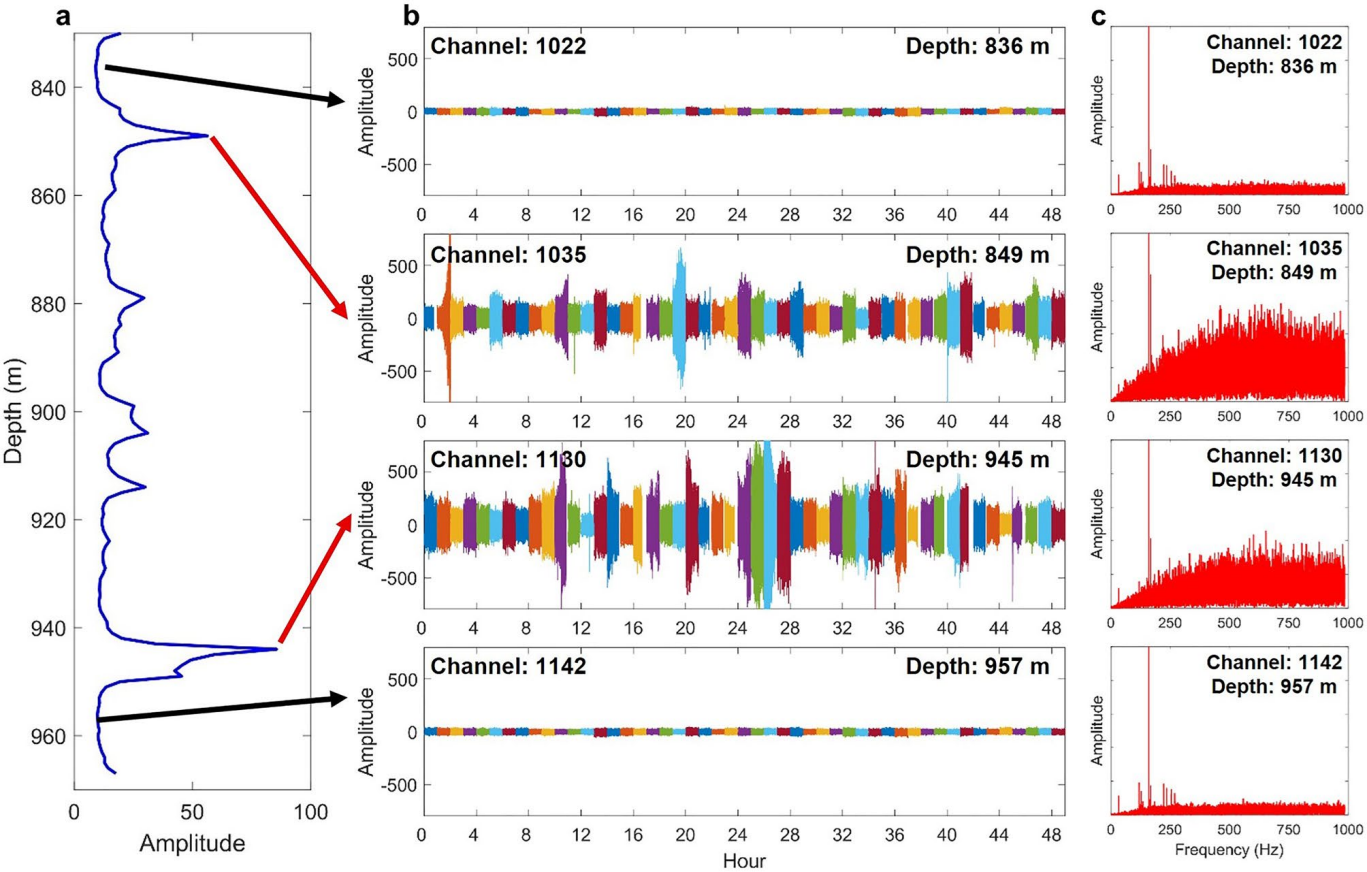
Comparing the average noise RMS profile with 48 h of data on different channels. (**a**) Average RMS depth profile of borehole DAS ambient noise. (**b**) 48 h of ambient noise traces with15-second segments for both quiet (channel 1022 and 1142) and noisy (channel 1035 and 1130) channels. Each segment starts at the 53^rd^ second of each hour. (**c**) Amplitude spectrums of 15 s noisy (1035 and 1130) and quite (1022 and 1142) traces.

We compare 48 h of borehole DAS ambient noise traces in both quiet and noisy zones of the DAS record in Fig. 7. For DAS channels 1022 and 1142, which are in the quiet zones of the RMS profile, we observe that the ambient noise seismic traces show small amplitudes with smooth and gentle variations over time. By contrast, DAS channels 1035 and 1130 coincide with two major peaks in the RMS profile, and show ambient noises with very large amplitudes as well as sudden and random variations over time. This drastic difference of noise amplitudes between the quiet and noisy zones at different depths may reflect the different properties of different formations.

To understand the frequency contents of borehole DAS ambient noise, we select a 15 s DAS record segment (UTC190429110053) and apply FFT to the noise to obtain noise amplitude spectra of DAS channels in both quiet and noisy zones (Fig. 7c). We select this DAS record segment for spectral analysis because it does not have Types B, C, and D noises. Although the two quiet channels are separated by approximately 121 m, their amplitude spectra show similar spectral features. Both amplitude spectrum plots in show peak amplitudes at a frequency of approximately 162 Hz. The amplitude spectra of two noisy channels also share similar features with peak amplitudes at a frequency of about 162 Hz. However, the amplitude spectra of noisy channels are more complex than those of quiet channels and contain more high-frequency contents. It is worth further investigating the significant frequency spectral differences between the quiet and noisy zones. These spectra differences may provide clues for the origin of the large amplitude noises.

**Figure 8:**
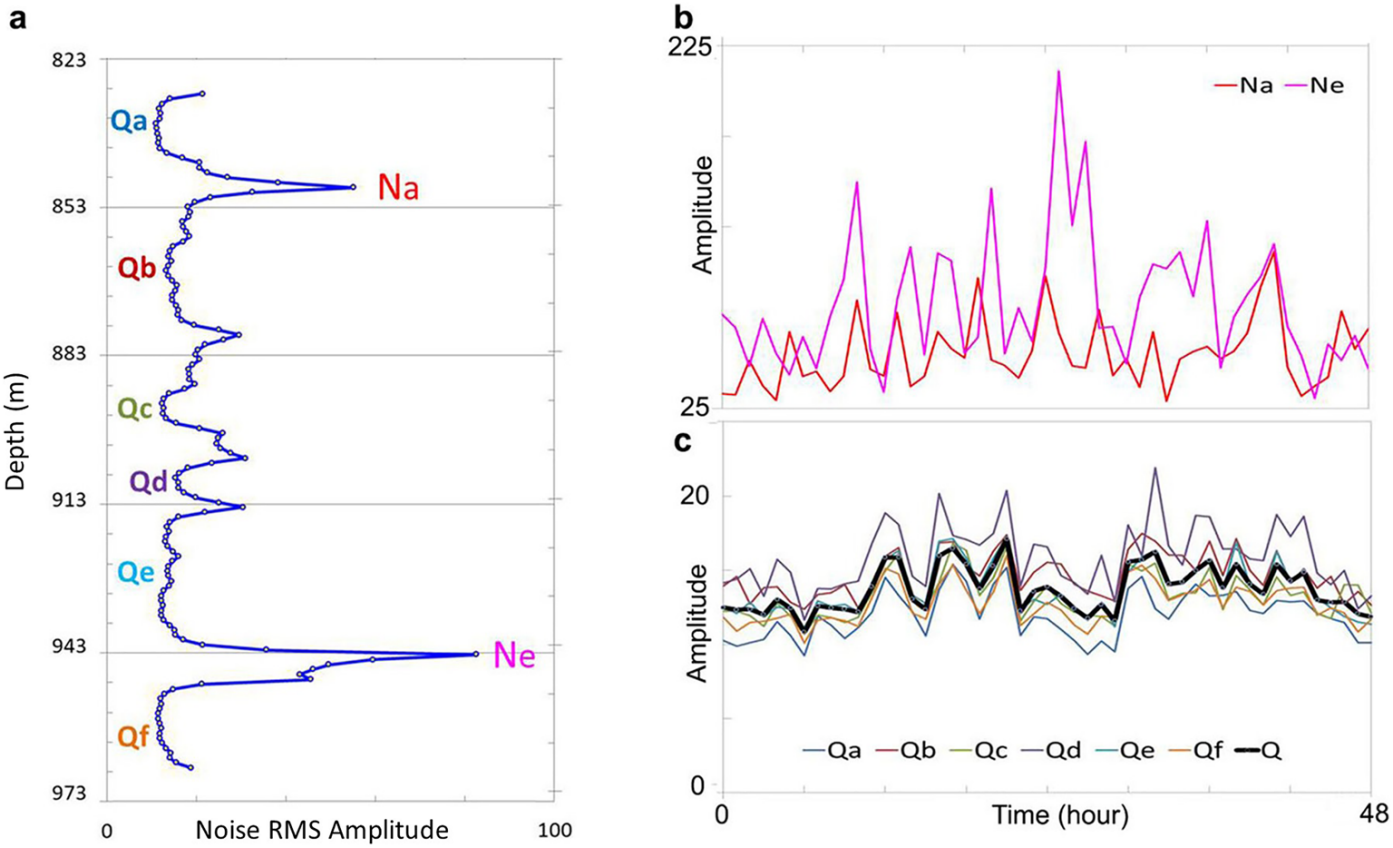
Noise amplitudes varied over time. (**a**) Average RMS depth profile of ambient noise marked with two major noise peak zones (Na and Ne) with two larger RMS peaks and six quiet zones (Qa to Qf) with smaller RMS values. (**b**) Ambient noise RMS amplitudes-time plots for the two channels with two larger RMS peaks (Na and Ne). (**c**) Noise RMS amplitude-time plots for the six quiet zones (Qa to Qf), averaged over each quiet zone. These six curves show the quiet and noise periods, which occur twice within 48 h, suggesting a possible daily variation. The thick black curve (Q) is an average over the six quiet zones, clearly demonstrating the possible daily variation pattern.

The noise RMS amplitude depth profile in Fig. 8a shows six quiet zones (Qa, Qb, Qc, Qd, Qe, and Qf) and five noisy zones with two major RMS amplitude peaks (Na and Ne) at depths of 849 and 945 m, respectively. We plot noise RMS amplitude temporal variations in a 48-h period for channels 1035 (849 m depth) and 1130 (945 m depth) in Fig. 8b. The maximum RMS amplitudes of the two noisy peak zones are approximately five to ten times larger than those of the quiet zones (Fig. 8c). We display the temporal variations of RMS amplitudes for the six quiet zones in Fig. 8c. These six curves are similar to one another and have only slight differences in RMS amplitudes. It is possible that daily variations occur in the quiet and noisy periods. The black curve Q in Fig. 8c is the average RMS curves of the six quiet zones, Qa ~ Qf. The daily variation pattern of noise RMS amplitudes in the quiet zones seems more apparent by observing this black curve. However, we need to analyze more DAS data recorded in the 14-day period to further test this hypothesis. In recent advances in DAS instrumentation, the DAS channel spacing can be as small as 0.2 to 0.25 m^18–20^. Therefore, such a DAS system can be used as a source-free DAS logging tool alongside conventional wireline tools in the near future.

## Data Availability

All borehole DAS ambient noise data from the Utah FORGE site are available at https://gdr.openei.org/submissions/1185.

## Code availability

Ambient noise analysis code is maintained by DL and is available on request with LANL’s approval to release.
